# Relationship between haemagglutination-inhibiting antibody titres and clinical protection against influenza: development and application of a bayesian random-effects model

**DOI:** 10.1186/1471-2288-10-18

**Published:** 2010-03-08

**Authors:** Laurent Coudeville, Fabrice Bailleux, Benjamin Riche, Françoise Megas, Philippe Andre, René Ecochard

**Affiliations:** 1 Sanofi pasteur, 2 avenue Pont Pasteur F-69367 Lyon cedex 07 France; 2 Hospices Civils de Lyon, Service de Biostatistique; CNRS, UMR 5558; Université Claude Bernard, Laboratoire Biostatistique Santé, Lyon, France

## Abstract

**Background:**

Antibodies directed against haemagglutinin, measured by the haemagglutination inhibition (HI) assay are essential to protective immunity against influenza infection. An HI titre of 1:40 is generally accepted to correspond to a 50% reduction in the risk of contracting influenza in a susceptible population, but limited attempts have been made to further quantify the association between HI titre and protective efficacy.

**Methods:**

We present a model, using a meta-analytical approach, that estimates the level of clinical protection against influenza at any HI titre level. Source data were derived from a systematic literature review that identified 15 studies, representing a total of 5899 adult subjects and 1304 influenza cases with interval-censored information on HI titre. The parameters of the relationship between HI titre and clinical protection were estimated using Bayesian inference with a consideration of random effects and censorship in the available information.

**Results:**

A significant and positive relationship between HI titre and clinical protection against influenza was observed in all tested models. This relationship was found to be similar irrespective of the type of viral strain (A or B) and the vaccination status of the individuals.

**Conclusion:**

Although limitations in the data used should not be overlooked, the relationship derived in this analysis provides a means to predict the efficacy of inactivated influenza vaccines when only immunogenicity data are available. This relationship can also be useful for comparing the efficacy of different influenza vaccines based on their immunological profile.

## Background

Influenza is a common, highly contagious viral respiratory disease. Annually it affects 5 to 15% of the world's population, causing considerable morbidity and mortality in all age groups [1]. Influenza vaccines have been available for more than half a century. For optimal efficacy, vaccine strain compositions are updated regularly to counter "antigenic drift" that occurs progressively from season to season as a consequence of immune selection, so that the vaccine antigens are as close as possible to the circulating wild-type antigens. Current inactivated vaccines comprise preparations of virus from two subtypes of influenza A (H1N1 and H3N2) and one of influenza B. Purification of these trivalent vaccines leaves mainly viral haemagglutinin (HA) and neuraminidase (NA) glycoproteins. The haemagglutination-inhibiting (HI) antibodies generated in response to stimulation by an exposure to HA prevents infection by disrupting the binding of the virus to host receptors. The concentration of HI antibodies in the blood (HI titre) is measured using a specific immunological assay [2].

Despite the extensive use of the HI assay in the annual approval process of inactivated vaccines [3,4] and in the evaluation of new seasonal or pandemic influenza vaccines, limited attempts have been made to use HI as a means to predict influenza vaccine efficacy. Based notably on the observations made in a seminal paper by Hobson et al [5], a HI titre of 1:40 is generally accepted to be associated with a 50% reduction in the risk of illness in a susceptible population [6], and can be referred to as the 50% protective titre (50% PT).

Recently, Gilbert et al. [7] used logistic regression to analyze the relationship between HI titre and vaccine efficacy but only as an illustrative example with data coming from one of the first clinical trials ever performed [8].

Better understanding of the relationship between HI titre and protection against illness may help evaluate vaccine efficacy when only immunological data are available. Pandemic vaccines offer a good illustration of circumstances in which an immune correlate is potentially useful for the assessment of vaccine efficacy [9]. More generally speaking, correlates of protection are valuable in any situation where practical issues or resource limitations prevent the direct estimation of vaccine efficacy.

Beyond the specific case of influenza, statistical validation of surrogate endpoints has generated extensive literature [10-13]. Recently, Qin et al [14] developed a framework for the identification of different levels of correlates of protection adapted to the context of vaccination. Several applications of this methodology exist for drugs in the literature (see e.g. Molenberghs et al [15]), but only a few can be found for vaccines using either the results of a single clinical trial [7,16,17] or simulated data [18].

Here we describe the development of a model, using a meta-analytical approach, that relates protection against laboratory-confirmed influenza to HI titre.

The methodological problems raised by the development of this model can be divided in three categories. The first category is related to the nature of the relation between HI titre and protection against influenza. This relation is unlikely to be of linear form and the preventive role of HI antibodies must be separated from other factors that influence the occurrence of influenza illness. The second stems from the geographic and temporal variation that affects not only virus circulation but also possibly the level of protection conferred by HI titres. Assessing such variations requires the use of a meta-analytical approach with datasets collected over time in vaccinated and unvaccinated populations. We therefore considered a nonlinear hierarchical model with random effects associated with all parameters to be estimated. The third type of methodological problem is directly linked to the nature of data available to perform this estimation. They were collected from articles published in the medical literature and in which data are presented for a limited number of HI titre intervals. The model developed therefore accounts for this interval-censorship.

## Methods

### A model for estimating the relation between HI titre and protection against influenza

#### A simple model for one study, no censorship and no covariates

Influenza illness is the result of a complex process involving the risk of being in contact with an infectious individual, the risk that this contact leads to infection and finally the risk that infection results in illness. Our objective is not to model this whole process in detail but to focus on the protection afforded to an individual by the level of humoral HI antibodies.

The model starts with a baseline risk (0 ≤ *λ *≤ 1) that an individual develops influenza in absence of any HI-related protection. To estimate the risk that an individual develops influenza (*P*(*y*_*j *_= 1)) in presence of HI antibodies, this baseline risk is combined with a function defining the contribution of HI titre to the individual's protection (0 ≤ *π *(*T*_*j*_, *θ*) ≤ 1, where T_j _is the HI titre and *θ *is the associated vector of parameters). More specifically:(1)

To fully characterize this estimation, the functional form associated with *π *(*T*_*j*_, *θ*), hereafter referred to as the HI-protection curve, needs to be specified. *π *(*T*_*j*_, *θ*) will be a flexible and smooth increasing function. In accordance with Dunning [16] it is specified as a two-parameter inverse logit function (*θ *={*α*, *β*}) applied to log-transformed HI titre values. The original model is then further modified to make its parameters more directly interpretable, leading to the following equation:(2)

The parameter *α *is closely linked to the 50% protective titre (50% PT) first identified by Hobson et al [5]: ∀*θ*, *T*_*j *_= *e*^*α *^⇒*π *(*T*_*j*_, *θ*) = 0.5. The parameter *α *can therefore be interpreted as a location parameter for the HI protection curve.

The parameter *β *is also easily interpretable since it is directly related to the slope of *π *(*T*_*j*_, *θ*) for , i.e. it describes the steepness of the HI protection curve.

#### A random-effects model with uncensored data with covariates

The inclusion of multiple datasets (i = 1, ..., I) enables to account for the heterogeneity likely to impact the HI protection curve. This was done using a hierarchical model, considering random effects for the parameters of the curve (*α*_*i*_, *β*_*i*_). The datasets considered for these random effects correspond to observations made for one virus strain in one study.

Part of this heterogeneity may be explained by relevant covariates. We tested the impact of several covariates by adding in some models a vector of binary variables X_i _with associated parameters *α*_*c*_, *β*_*c*_. This representation limits the analysis to covariates whose values are common to all subjects of a given dataset and are consistent with the type of data considered.

Finally, the baseline risks *λ*_*i*_, which are unrelated to the HI protection curve, will be treated as independent dataset-specific parameters. No assumption was therefore made on the variation of *λ*_*i *_across studies. Similarly to random effects, we consider baseline risks to be specific to datasets corresponding to observations made for one influenza strain in a study.

The probability to be estimated becomes(3)

Where *θ*_*i *_= {*α*_*i*_, *β*_*i*_, *α*_*c*_, *β*_*c*_}.

#### A random-effects model with interval-censored data with covariates

Censorship does not modify the structure of the random-effects model but rather necessitates an additional stage for its estimation. In a Bayesian framework, censorship can be accounted for using data augmentation techniques [19]. The basic idea is to consider the unknown T_ij _values as latent variables and to use sampling to impute their possible values given their underlying distribution and the interval to which they are known to belong . With this approach, T_ij _are in fact treated as additional parameters. We considered here that the log-transformed HI titres are normally distributed (with mean *μ*_*i *_and standard deviation *σ*_*i*_) and specific to each dataset considered in the analysis. Provided that censorship is non-informative, the relationship between HI titres and clinical protection continues to be estimated using equation (3).

### Estimation methods

**Figure 1:**
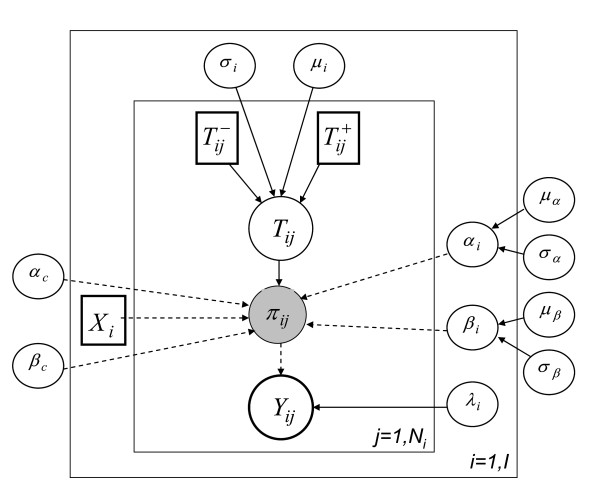
**Directed acyclic graph of the interval-censored model with covariates**. Square boxes represent fixed quantities, white circles stochastic nodes, grey circles logical nodes, solid arrows stochastic dependencies and dashed arrows deterministic dependencies. Bold lines corresponds to nodes associated with data.

The nature of the model considered led us to adopt a Bayesian approach to perform the estimation of its parameters. Markov Chain Monte Carlo (MCMC) methods were implemented using the Bayesian software package WinBUGS [20]. The corresponding directed acyclic graph is presented Figure 1. Posterior summary statistics were based on 3 Markov chains of 20,000 lengths after a burn-in period of 20,000 iterations. Convergence was assessed using Gelman-Rubin statistics [21] as well as the iteration history and kernel densities.

We assumed the two random parameters of the HI protection curve to be normally distributed (). For dataset-specific and other parameters, we selected the following non-informative prior distributions:

Our choice of non-informative priors is quite usual. It is however worth mentioning that for the baseline risks *λ*_*i*_, we specifically considered Jeffreys prior for the Beta distribution which can be regarded as less informative than the alternative choice *λ*_*i *_~ *Beta*(1,1) (see e.g. Gupta and Nadarajah [22]).

The comparison of the estimation of the HI protection curve obtained with the different models tested was mainly performed using the Deviance Information Criterion (DIC) [23]. This criterion, an Akaike-like criterion for Bayesian models, assesses the goodness-of-fit of a model using posterior mean deviance. To compare models with and without covariates, we also used the 95% credible interval of the parameters associated with covariates.

### Data

To estimate HI-related protection, we reviewed the literature to identify datasets combining information on HI antibody titre and the occurrence of influenza. Relevant publications from 1945 to 2006 were identified in the Medline and Embase databases using the search terms: influenza, immune correlate, protection, vaccination, vaccine, immunogenicity, protective efficacy, serological surrogate.

We identified 36 articles, 21 of which were excluded based on inclusion of non-target populations (children in 9 studies; pregnant women in 1 study); insufficient data to allow quantification of the link between HI titre and influenza protection (6 studies); vaccination using live vaccines (3 studies); previously reported data (2 articles).

Fifteen articles were thus retained: 6 challenge studies, 5 clinical trials and 4 cohort studies (Table 1). In challenge studies, healthy adult volunteers were randomised to receive vaccine or placebo and then were exposed to a fixed dose of influenza virus after a pre-challenge serum HI titre assessment. In clinical trials, participants were randomized to receive vaccine or placebo before the influenza season, and had at least a pre-season serum HI titre assessment. In cohort studies, all the participants included in the cohort had at least a pre-season serum HI titre assessment, performed after immunization for vaccinated participants. In all clinical trials and cohort studies, the occurrence of influenza was observed during the influenza season following the HI titre assessment. Most study subjects were adults younger than 60 years, and only one study included elderly adults (60+ years) [24].

**Table 1:** Characteristics of studies included in the analyses

Author, year	Design	Vaccination status^£^*			Vaccine strain	Number of HI titre intervals^$^	Drop-out rate#
		Vaccinated	Unvaccinated	Unknown			
Bell et al., 1957 [32]	Challenge	32 (14)	-		A	3	> 10%

Clark et al., 1983a [33]	Challenge	49 (7)	23 (16)		A	6	≤ 10%

Clark et al., 1983b [34]	Challenge	39 (6)	58 (23)		A	5	≤ 10%

Dowdle et al., 1973 [35]	Trial	227 (90)			A	4	≤ 10%

Eaton and Meiklejohn, 1945 [36]	Trial	94 (4)	145 (9)		A(Olson)	5	≤ 10%
		94 (9)	145 (4)		A(PR8)		
		94 (9)	145 (4)		B		

Evans, 1975 [37]	Cohort	696 (262)	-		A	5	NA
		346 (47)			B		

Farnik and Bruj, 1966 [38]	Cohort	-	248 (52)		A	5	≤ 10%

Fox et al., 1982 [39]	Cohort	-	-	222 (13)	A(H1N1)	4	≤ 10%
				343 (98)	A(H3N2)	3	
				140 (28)	B	3	

Goodeve et al., 1983 [40]	Challenge	97 (9)	23 (15)		B	6	≤ 10%

Greenberg, Couch and Kasel, 1974 [41]	Cohort	-	212 (97)		A	5	≤ 10%

Hirota et al., 1997 [24]	Trial	84 (4)	118 (9)		A(H1N1)	3	≤ 10%
		84 (4)	118 (9)		A(H3N2)		
		84 (4)	118 (9)		B		

Hobson et al., 1972 [5]	Challenge			106 (20)	A(Field Trial)	9	NA
				345 (166)	A(HK Salisbury)		
		-	-	119 (62)	A(Pre-1968)		
				462 (135)	B		

Meiklejohn et al., 1952 [42]	Trial	-	101 (5)		A	6	> 10%

Potter et al., 1977 [43]	Challenge	134 (26)	-		A	6	≤ 10%

Salk et al., 1945 [8]	Trial	82 (2)	246 (21)		A(Weiss)	10	> 10%
		82 (2)	144 (12)		A(PR8)		

*: Number of observations (number of influenza cases)

$. Number of reported intervals on pre-season HI titres.

£ Vaccination performed just before exposure

# Number of subjects analyzed/Number of subjects included in the analysis

Several studies reported data for both vaccinated and non-vaccinated populations and HI titres corresponding to different vaccine strains. We split this information into different datasets to consider homogeneous groups of subjects in terms of HI titre information and vaccination status. Thus, the 15 articles provided 37 datasets presented Table 1 for a total of 5889 observations and 1304 influenza cases. All except 39 influenza cases in one study [24] were laboratory confirmed using paired serology (four fold change) or virus isolation.

These data enabled us to test the effect of circulating strain (A or B), vaccine exposure (yes or no), study design (clinical trial, challenge study or cohort study) and diagnostic method (laboratory-confirmed clinical diagnosis, serological diagnosis and clinical diagnosis).

HI titres are measured using a two-fold serial dilution assay, which determines the highest dilution of serum which still inhibits haemagglutination [2]. So while the concentration of HI antibodies can be considered to be a continuous variable, and will be treated as such in our analysis, the assay only provide a limited number of possible results, creating the first level of censorship. A second level of censorship results from how the data are published. In most of the cases, data are reported for a limited number of HI titre intervals and not for each possible result of the HI assay.

## Results

**Figure 2:**
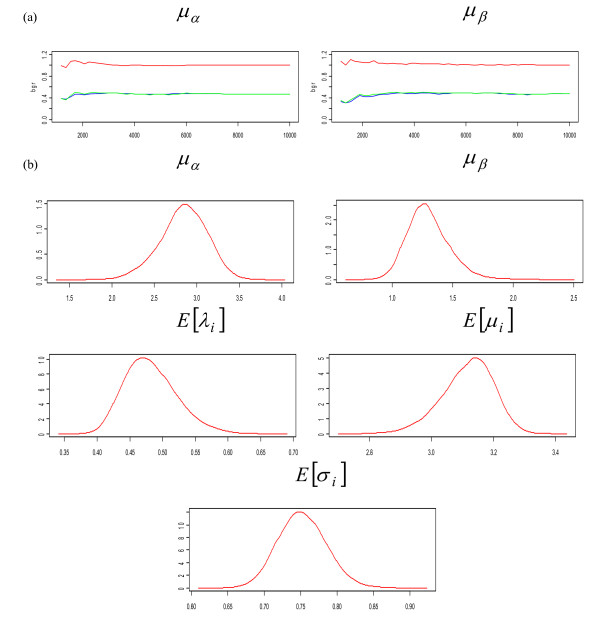
**Gelman-Rubin diagnostic plot (a) and kernel densities (b) (ALL model)**. GR plot: blue corresponds to within-chain variability, green to between chain variability and red to their ratio.

**Figure 3:**
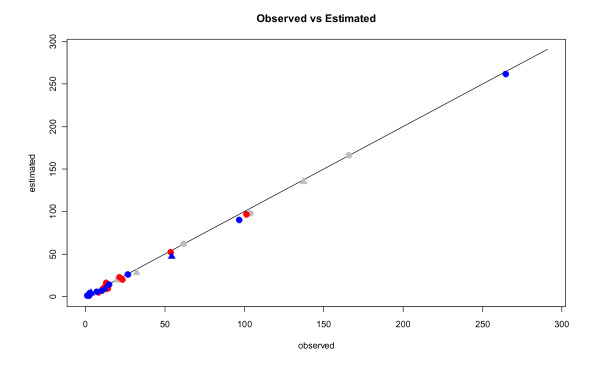
**Estimated (Y-axis) versus observed (X-axis) number of influenza cases for the 37 datasets considered in the analysis (ALL model)**. Red corresponds to vaccinees, blue to non vaccines, grey to unknown status, circles to type A strain and triangles to type B.

Gelman-Rubin statistics and kernel densities (Figure 2) confirm that the markov chains and the burn-in period are sufficiently long for the posterior statistics to be meaningful. The results presented Table 2 show a strong and positive relationship between HI titre and the occurrence of influenza: *β *is significantly different from 0 and positive whatever the model considered. These results are reinforced by the overall good fit to the data illustrated Figure 3 for the ALL model (all available data included and no covariate).

**Table 2:** Impact of vaccination status and vaccine strain on the estimation of HI-related protection:

Model name^$^	ALL	STRAIN	ALL_V	VAC	HOB
Number of subjects	5899	5899	4162	4162	1032
Number of flu cases	1304	1304	782	782	383

Parameter Estimates*					
***μ*_a _[95% CI]**	**2.844 [2.25;3.36]**	**2.964 [2.27;3.53]**	**2.344 [1.49;3.14]**	**2.615 [1.63;3.56]**	**3.385 [1.53;5.27]**
*σ*_a _[95% CI]	0.845 [0.44;1.41]	0.85 [0.42;1.43]	0.944 [0.33;1.69]	1.06 [0.37;2]	1.415 [0.28;5.37]
***μ*_b _[95% CI]**	**1.299 [1;1.69]**	**1.404 [1.04;1.91]**	**1.061 [0.77;1.48]**	**1.205 [0.81;1.69]**	**2.102 [-0.27;6.36]**
*σ*_b _[95% CI]	0.376 [0.1;0.76]	0.386 [0.07;0.81]	0.275 [0.05;0.66]	0.206 [0.01;0.62]	2.102 [0.19;11.93]

α_c _[95% CI]		-0.467 [-1.72;0.81]		-1.04 [-2.93;0.7]	
*β*_c _[95% CI]		-0.305 [-1.06;0.42]		-0.377 [-0.98;0.23]	
E[*λ*_i_] [95% CI]	0.482 [0.41;0.57]	0.481 [0.41;0.57]	0.497 [0.39;0.61]	0.531 [0.41;0.67]	0.726 [0.64;0.81]
E[*μ*_i_] [95% CI]	3.116 [2.93;3.26]	3.117 [2.94;3.26]	3.129 [2.91;3.3]	3.13 [2.91;3.3]	3.157 [2.96;3.35]
E[*σ*_i_] [95% CI]	0.752 [0.69;0.82]	0.752 [0.69;0.82]	0.775 [0.71;0.85]	0.773 [0.71;0.85]	0.181 [0.16;0.21]

**DIC**	**4667.0**	**4667.0**	**3074.0**	**3077.0**	**964.2**

$. ALL: All datasets and no covariate, STRAIN: All datasets + 1 covariate for virus strain (reference category: type A virus), ALL_V: datasets with information on vaccination status, VAC: datasets with information on vaccination status + 1 covariate for vaccination status (reference category: vaccinated), HOB: data reported in Hobson et al. [5], no covariate

* Reported values for each parameter: posterior mean value and 95% credible interval

The impact of covariates related to vaccination status and virus strain are important for the interpretation of the HI protection curve. In both cases, their impact is not significant: DIC does not decrease or even increase when {*α*_*c*_, *β*_*c*_} are added to the model and none of these parameters significantly differ from 0. The same curve therefore seems to apply regardless of the type of virus strain considered and regardless of the vaccination status of the subjects. This latter result is of particular importance as the absence of significant differences between vaccinated and non vaccinated subjects corresponds to a condition set by Prentice [10] for defining an accurate surrogate endpoint.

We also estimated the HI protection curve considering only the data reported by Hobson et al [5] (HOB model). The 50% level of protection is obtained with this model for a log-transformed HI titre is 3.38 [95% CI:1.5;5.2] corresponding to 1:29 in the natural scale, which is consistent with the 50% protection titre identified by Hobson and his co-workers (1:18-1:36) but remains higher to that obtained in the ALL model (1:17 in the natural scale). The credible interval is also much wider in the HOB model [1:5;1:195] than in the ALL model [1:10;1:29], which indirectly supports the use of a meta-analytical approach to obtain more robust estimates and to distinguish uncertainty from heterogeneity.

The standard deviations  reported in Table 2 provide insight on the importance of the heterogeneity of the HI protection curve across studies. Significant variations can be observed: the 25% percentile associated with the variation of the 50% PT from one study to another can be calculated to correspond to a HI titre of 1:10 and the 75% percentile to a HI titre of 1:30. Similarly, the steepness of the HI protection curve, as measured by *β*, ranges from 1.05 to 1.55.

Finally, we tested covariates corresponding to the conditions in which the observations on influenza were collected (Table 3): study design (DES model) and diagnostic method (DIAG model). None of these covariates improved data fit as measured by the DIC criterion or the significance of the corresponding parameters.

**Table 3:** Impact of study design and diagnostic method on the estimation of HI-related protection

Model name^$^	ALL	DES	DIAG	DOR
Number of subjects	5899	5899	5899	3825
Number of flu cases	1304	1304	1304	612

Parameter Estimates*				
***μ*_*α *_[95% CI]**	**2.844 [2.25;3.36]**	**2.55 [1.49;3.44]**	**2.751 [1.79;3.75]**	**1.594 [1.78;6.39]**
*σ*_*α *_[95% CI]	0.845 [0.44;1.41]	0.936 [0.49;1.56]	0.905 [0.45;1.54]	0.956 [0.23;1.88]
***μ*_b _[95% CI]**	**1.299 [1;1.69]**	**1.222 [0.8;1.77]**	**1.181 [0.75;1.72]**	**-1.414 [3.59;1.37]**
*σ*_*β *_[95% CI]	0.376 [0.1;0.76]	0.412 [0.1;0.85]	0.428 [0.14;0.83]	0.211 [0.02;0.53]
*Study design*^*a*^				
*α*_co _[95% CI]		0.096 [1.23;1.44]		
*β*_co _[95% CI]		-0.021 [0.79;0.78]		
*α*_ch _[95% CI]		0.577 [0.82;1.97]		
*β*_ch _[95% CI]		0.241 [0.54;1.09]		
*Diagnosis*^*b*^				
*α*_ser _[95% CI]			0.091 [1.57;1.54]	
*β*_ser _[95% CI]			0.131 [0.58;0.89]	
*α*_ili _[95% CI]			0.07 [1.65;1.36]	
*β*_ili _[95% CI]			0.415 [0.52;1.45]	

E[*λ*_i_] [95% CI]	0.482 [0.41;0.57]	0.5 [0.43;0.59]	0.491 [0.42;0.58]	0.505 [0.38;0.62]
E[*μ*_i_] [95% CI]	3.116 [2.93;3.26]	3.115 [2.93;3.26]	3.112 [2.94;3.26]	3.168 [2.96;3.33]
E[*σ*_i_] [95% CI]	0.752 [0.69;0.82]	0.751 [0.69;0.82]	0.751 [0.69;0.82]	0.809 [0.74;0.89]

**DIC**	**4667.0**	**4670.0**	**4669.0**	**2623.0**

$. ALL: All datasets and no covariate, STRAIN: All datasets + 1 covariate for virus strain (reference category: type A virus), ALL_V: datasets with information on vaccination status, VAC: datasets with information on vaccination status + 1 covariate for vaccination status (reference category: vaccinated), HOB: data reported in Hobson et al. [5], no covariate

* Reported values for each parameter: posterior mean value and 95% credible interval

**Figure 4:**
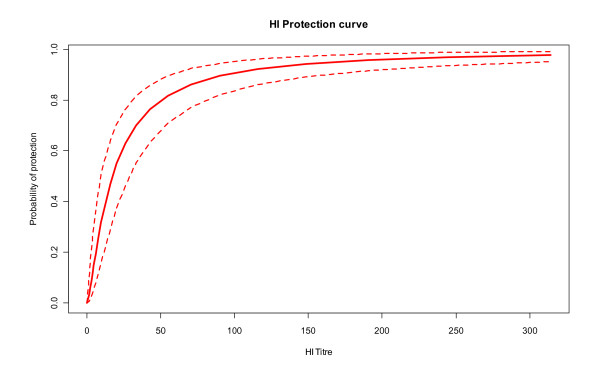
**Estimated probability of protection according to the level of HI titre**. (All Model - Posterior Mean value and 95% credible interval).

Overall, our results support the conclusion that the best representation of the relationship between HI titres and protection against influenza is obtained when combining all available data without any covariate (ALL model). The corresponding HI protection curve is presented Figure 4.

## Discussion

Although the association between serum HI antibody titre and protection against clinical influenza is well established [25,26], the precise nature of the relationship has drawn little attention. To address this, we developed a model to provide an estimate of the level of protection associated with any HI titre level for use to predict influenza vaccine efficacy. Using HI data from multiple studies, the model showed a positive and significant relationship between immunogenicity and clinical protection. The relationship was notably found to be similar for vaccinated and unvaccinated subjects which is in accordance with Prentice criterion for a good surrogate marker [10].

Our results challenge the usual approach of defining protection based on the identification of a single threshold. The slope of the protection curve in all of the models we tested favoured a progressive increase in protection with increasing HI titre rather than a discrete threshold that could be applied to each individual. The incremental increase in clinical protection is however particularly important at titres of up to 1:100 (which includes the commonly used 1:40 threshold). Additional benefits become marginal beyond 1:150, which concords with the result derived by De Jong et al [25] who estimated the median 90% PT for influenza vaccines to be 1:192. Our analysis therefore supports their conclusion that developing influenza vaccines capable of reducing the number of poor or low responders would be clinically beneficial.

The comparison between our reference case combining all available data (ALL model) and the model based only on data reported by Hobson et al (HOB model) provides a good illustration of the added value of the analysis presented here. The results of HOB model are consistent with the results reported in the original paper in terms of 50%PT. Our model therefore does not contradict previous results, but provides an enhanced representation of the relationship between HI titre and protection against influenza. In addition, the large confidence intervals associated with HOB model highlights the need to combine a large number of observations to get an accurate representation of this relationship and to account for the heterogeneity across studies. This is precisely the added value of the meta-analytical approach proposed here. This meta-analysis confirms the existence of diversity likely to affect the interpretation of the results of a single study. This heterogeneity can be explained both by the conditions in which the subjects were exposed to influenza (e.g. time between HI titre assessment and occurrence of influenza) or by the design of the studies (e.g. laboratory methods).

It has to be mentioned that to simplify model specification, we considered random effects capturing jointly the heterogeineity across studies and across virus strains in the same study. A more detailed representation would have been possible notably to be able to consider specifically these two levels of heterogeneity.

Overall, the different covariates considered did not improve the fit to the data either measured by the DIC criterion or by the significance of the corresponding parameters. This result holds not only for vaccination status but also for the type of viral strain, study design and diagnostic method. This supports the idea of the large applicability of the HI protection curve derived in this analysis.

Some similarities can be found between our results for HI assay and a statistical Surrogate of Protection (SoP) defined by Qin et al [14] i.e. an immunological measurement characterized by a relationship with the end point of interest (in this case laboratory-confirmed influenza) that is similar in vaccinees and non vaccinees. One important point is that no attempt was made to establish a causal relationship but only to identify a statistical link. it can for instance be argued that HI antibodies only relate to the humoral immune response and therefore neglects the role played by cell-mediated immunity [27]. However, the coexistence of different biological mechanisms does not preclude the identification of a statistical link with one specific measurement. It only requires that this link is not improperly interpreted as sufficient to explain the complex biological mechanisms that trigger the protection against an infectious disease.

The main application of a surrogate of protection is predicting vaccine efficacy. Although our analysis was at this stage only focused on the derivation of an HI protection curve, this is clearly an important next step. The HI protection curve can also be used for comparing vaccines characterized by different immunological profiles. This can be seen as an improvement over the use of standard criteria such as seroprotection rates (i.e. percentage of subjects with a HI titre above the 1:40 threshold for protection). As pointed out by Nauta et al. [28], such criteria may be misleading for this type of comparison if the HI protection is better described as a curve than using a threshold approach.

The reliability of our model depends directly on the quality of data used for the estimation and calculation phases. As we used published data, there were some limitations that could not be overcome. The data were acquired and published over a period of many years, and the studies involved heterogeneous populations, different study designs, and in some cases inadequate or no description of randomization procedures. The HI test itself changed over time and is also subject to inter-laboratory variability [29]. Other differences noted were: vaccine composition and dosage, case definition, interval between vaccination and antibody titration, assay method. The selected datasets also had limited information on the status of confounding factors such as pre-vaccine antibody level titre, influenza vaccination history, prevalence of co-morbidities, nutritional deficiencies, chronic exposure to stress or drugs that could affect the immune system [30]. The lack of available information on baseline covariates such as previous history of influenza disease or vaccination is another limitation. Antigenic similarity between vaccine strains and circulating strains, which vary with time, even during a single season, plays a key role in vaccine efficacy. While our use of results from 15 studies may have partially overcome this heterogeneity, reported immunogenicity results generally correspond to vaccine strains having a good match with circulating strains. This problem of matching is particularly critical for pandemic vaccines, and the direct application of our results to vaccines developed in the context of pandemic preparedness should be considered very cautiously. It is also important to stress than H5N1 vaccines require a different HI test than the one used for seasonal vaccines [31].

To further evaluate and establish this model, an important development will be to perform a similar analysis using data that includes detailed information at the individual level with a virological diagnosis of influenza (most cases considered in this analysis were serologically confirmed). The accuracy of such an analysis will however ultimately depend on the number of influenza cases considered. We believe that our use of over 1000 influenza cases in establishing our model as well as the consideration of study heterogeneity can be seen as the major strength of the analysis performed.

Finally our analysis was exclusively focused on the case of influenza. However, the question raised by the identification of a good correlate of protection is applicable to all vaccine-preventable diseases. Meta-analytical approaches have been extensively used for validating surrogate endpoints for therapeutic drugs [15], but the number of applications to vaccines remains very limited. The approach used here, which relies on published information to access a large number of cases (the "price to pay" in terms of data quality being here censorship), could be easily adapted to other vaccine-preventable diseases.

## Conclusions

The model developed enables us to specify the relationship between HI antibody titres and clinical protection against influenza while accounting for heterogeneity among studies. This relationship appears consistently positive and similar irrespective of vaccination status or viral strain and could be used to predict the efficacy of inactivated influenza vaccines when only immunogenicity data are available.

## Competing interests

LC, PA, FB and FM are employees or were employees of sanofi pasteur at the time the manuscript was prepared.

## Authors' contributions

LC performed the statistical analyses, contributed to the definition of the methodology and the writing of the manuscript. FB contributed to the definition of the methodology and was involved in writing the manuscript. BR was involved in the definition of the statistical methodology. FM performed the literature review and was involved in writing the manuscript. PA contributed to the literature review, to the definition of the methodology and the writing of the manuscript. RE contributed to the definition of the statistical methodology and the writing of the manuscript. All authors have read and approved the final manuscript.

## Pre-publication history

The pre-publication history for this paper can be accessed here:

http://www.biomedcentral.com/1471-2288/10/18/prepub
